# Dr. Moises's Case of Needles extracted

**Published:** 1802-01

**Authors:** Hugh Moises


### To Dr. BRADLEY.

Dear Sir,

In giving the following Case, I faithfully transcribe the minutes taken by the physicians and surgeons of the General Hospital, Nottingham, under whose immediate care the patient (Kate Hudson) was received at her different admissions. As at some periods of her disease, frequent consultations were held, the minutes were more carefully made and preserved at those periods; while at others, when the symptoms were considered as less urgent, little, or very unsatisfactory, notice is taken of them. To this may be added, the unfortunate loss of one of the books, to which I was in consequence excluded a reference at the time I collected the case into my Place-book.

I had formerly mentioned the outlines of the case to many professional men, who held deserved rank in the medical world; and of whom I might say, with Cicero, that they were

"Interiores et reconditæ literæ."

I, however, found that the tide of skepticism might hurry me into endless controversy, without a prospect of any practical advantage being derived from the discussion. With these impressions I hesitated to make the case public (as it was my intention to have done at the Lyceum Medicum, in 1792) well knowing that

"Philosophi ætatem in litibus conterunt."

Nor do I know whether the more general candour which prevails among medical men at this time, in the reception of unique cases, will lead you to admit this into the Medical Journal, neither am I anxious to press its introduction; if rejected, it may add to your own private collection. Suffice it to say, in respect to the facts it contains, that Dr. Storer, Mr. Wright, Mr. Bigsby, Mr. Attenborrough, Mr. Thompson, and every other professional man in the town of Nottingham, who were resident there at the time, are in full possession of the case, and are at liberty to confirm or contradict any part of my statement, according as I may be found correct or otherwise. I believe it was the intention of Doctor Snowden White, then senior physician to the Hospital, to have published the case; but his premature death from phthisis hæmoptoïca, I suppose, prevented his carrying his intention into execution. It has, therefore, hitherto been but lamely communicated to a few practitioners; nor can I detail it in the manner I could wish, for reasons already given.

 The

The preſerved facts of the caſe are before you; and for their being facts, I might pledge myſelf to you in the language of Terrence,

"Liquet mihi dejerare."

I have the honour to be, &c.

*16th Nov.* 1801. HUGH MOISES, M. D.

---

KATE HUDSON, a ſingle woman, aged 31, was admitted into the General Hoſpital, Nottingham, on the 4th of Auguſt, 1783, for an inflammatory affection of the right arm; her uſual occupation had been that of ſweeping the pews and aiſles of a church.—On inſpection of the arm two needles were diſcovered under the ſkin, a little above the dorſal ſide of the wriſt. They lay in a tranſverſe direction, and were readily extracted by puſhing the points through the ſkin and laying hold of them with a pair of forceps. Upon a more minute examination, ſome more needles were felt about three inches higher up the fore arm, but farther back than the others, and more over the flexor muſcles; theſe lay longitudinally, and appeared to have their heads downwards. Theſe needles were extracted as before, a ſmall puncture with a lancet having been previouſly made. (Of the number of the laſt-mentioned needles, I find no mention.)

Aug. 6th. Another needle is felt very plainly a little below the former place.

7. The nurſe, in attempting to extract one from her leg, has broken it, and left part of it in. A large needle has been taken away from her foot, which was laying acroſs her inſtep, and among the tendons.

Oct. 11. A very large darning needle was this day extracted from her right breaſt, ſeemingly buried within a part of the gland; thinks ſhe feels another needle very deep ſeated under the gland in the middle of the breaſt; complained of great pain in the breaſt after the removal of the needle, which, in about an hour afterwards, became ſo exceſſive as to throw her into convulſions.

℞. Tinct. fœtid ʒvj. aq. puleg. ſimp. ℥ij. tinct. theb. gt. lx. M. capt. ℥ſs. ſtatim et repet. omn. dimid. hor. durante paroxyſm. hyſteric.

Nov. 1ſt. The convulſions have continued at periods till now; the needle ſtill appearing to lie very deep within the breaſt; and about three days ago her jaws became locked; very weak and low; pulſe ſmall and weak; made an inciſion quite through the breaſt, and extracted a large needle which adhered to the tendinous faſcia covering the pectoral muſcle; afterwards brought the lips of the wound together by adhesive plaisters. (From this last report I find none other until)

Jan. 12th, 1784. Has now very severe pains in the breast; apply Goulard's fomentation sprinkled with laudanum. Capt. mist. cathat. ℥iss. pro re nata.

15th. Took her purging medicine last night, which had no effect; fomentation increased the pain; omit it.—℞. Infus. fenn. com. ℥v. tinct. fenn. ℥ij. jalap ʒij. tart. solubilis ʒij. ft. Mist. aperient. capt. ℥jss. cras mane hora 7ma. Touch the fungous ulcers with the weak solution of lunar caustic.

19th. ℞. Pulv. cort. peruv. rubr. ℥j. capt. ʒj. hora 7ma. et 11ma. mane in lactis vaccinæ.

24th. Omitt. pulvis.—℞. Infus. cort. peruv. frigid. ℥x. tinct. cort. peruv. c. ℥ij. M. capt. ℥jss hora xima. mane et 4ta. P. M.

27th. Infusion does not agree; omit it.—℞. Ext. cort. peruv. ʒj. ft. pil. xij. capt. ij. hora 11ma. mane et 4ta. P. M.

31st. Pills disagree; breast healing; and a needle now perceptible on the interior side of the breast.—℞. Ext. cicutæ ʒss. ft. pil. xvj. capt. j. ter in die, dos. augend. pro re nata.

Feb. 3d. Passed a pin yesterday by urine, which was not coated, or particularly corroded; and this day, with the same excretion, passed a needle.—Solut. g. arab. c. pro potu commune. Had much pain in the sphincter vesicæ before and during the passage, and the urine bloody, though less so to day.—Cont. pil. modo capt. jss. hor. somn.

5th. Passed another needle yesterday by urine; is faint and low.—Omit. pil.—℞. Julep. cardiac ℥vj. tinct. theb. gt. xl. M. capt. ℥j. aggrediente languore et repet. pro re nata.

11th. The breast healing fast, less pain, and the needle not easily distinguishable; has had pain in the throat and vomiting, which brought up blood; feels still an obstruction in swallowing.—Cont. solut. g. arab. et mist. cardiac, pro re nata.

12th. Brought up a needle by vomiting.—Cont. potus commune.

14th. Had a needle extracted from her breast.

16th. Complains of pain in the lower part of the thorax, betwixt the ribs, but nothing to be felt.

19th. Pain now in her breast, as when a needle was there before; that between the ribs gone off.

23d. Much as she was.—℞. Julep. cardiac. ℥vj. tinct. theb. gt. 80. M. capt. ℥j. aggrediente languore vel urgente dolore.

26th. Two days ago the whole of the breast began to be inflamed, and the inflammation continues.—Capt. mist. purg. statim. Cont. solut. g. arab. pro potu.

March 3d. Inflammation gone off.—Cont. pot. commune, pro re nata.

8th. Still complains of pain deep feated in her breaft, which prevents her refting.—Capt. julep. card. anodyn. ℥j. hora fomn.

11th. Pain ftill continues.—Capt. julep. card. ℥fs. o. n. h. f. aa. tinct. theb. gt. xxv. augente dos. pro re nata.

16th. Pain continues.—Takes tinct. theb. gt. c. Cont. pro re nata.

19th. The needle in the breaft to be extracted.

22d. It paffed into the thorax during the operation; part of the gland, which was fchirrous, was removed.—Capt. julep. anod. urgente dolore.

26th. No pain from the needle; breathing eafy.—Cont. med. pro re nata.

29th. Felt the needle in her ftomach, and threw up a confiderable quantity of congealed blood.

April 5th. Has had no pain from the needle fince laft report; breaft healing.

8th. Continues mending; no fymptom of any more pins or needles.

26. Quite well; difmiffed cured.

May 7. Is re-admitted; about a week fince was taken with pain, a pricking fenfation at the ftomach, and vomited a matter which confifted of a folution of a pin fimilar to what fhe paffed by urine, with fome ftreaks of blood, attended with external inflammation, with two fmall ulcers, which are now much better.—Ex ungt. alb. vel neutral. empl. applic. Ordered common diet, with folutio g. arab. c. pro potu commune fumat. aq. mephit. alk. ℥j. ftatim et omni 5ta. hora repet. fuperbibend. fucci limoni ℥fs.—℞. Tinct. theb. gt. xx vel xxx. hora fomni fumend. et gt. x. repetend. pro re nata urgente dolore, fi vomitu rejicitur tinct. theb. capt. extract. opii gr. j. vice.

10th. Laft night took tinct. theb. gt. lxxx. Procured no reft; complains of great pain in the ftomach, and thinks fhe feels two or three pins or needles, and that they change their pofition.—Capt. tinct. theb. gt. xx. urgente dolore et repet. pro re nata.—℞. Decoct. tamarind ℔j. manna ℥j. M. capt. q. s. in die ad folut. an. alv.

11th. Brought up three pins, two corroded, one not much fo.

13th. ℞. Empl. ex. ammon. cum merc. ʒvj. opii, camphoræ aa ʒj. M. ft. empl. later. dolent. applicand.

17th. Plaifter taken off; pain was relieved by it; but the part fore and a little ulcerated; ftomach yet fore.—Cont. med.

21ft.

21ſt. Yeſterday threw up matter ſimilar to what came from her ſtomach when the pins were there; complains of much pain in her ſtomach, but no pricking feel, except when preſſed; feels as if matter diſcharged from the part into the ſtomach; jaw locked, and a little ſubſultus tendinum.—Capt. tinct. theb. gt. xxx. in pauxil. ſolut. g. arab. c. et repet. pro re nata.—℞. Ungt. merc. fort. ℥j. camphor. tinct. theb. aa. ʒj. M. ft. lin. fricand. ſcrob. cord. et fauces ter 4terve in die. Ordered milk, whey, butter-milk and water, and weak broths frequently for diet.

22d. Had very little reſt during the night; early in the morning got up and took three tea-ſpoons full of tinct. theb. without any ſenſible effect; had frequent ſpaſms in the courſe of the day; went into the warm bath this evening; was very faint and much convulſed on coming out, afterwards conſiderably better; took the following bolus about eight o'clock. ℞. Calomel gt. x. opii, gr. j. ft. bolus.

23d. Has had a tolerable night's reſt; bolus has not purged her; thinks herſelf much eaſier; no relaxation of the jaw; repet. bolus ſtatim ſine opio; has much pain and ſubſultus tendinum; repet. baln. tepid. et bol. cum opio par ſomn. augente ſubſult. tend. capt. tinct. theb. gt. lx. et repet. pro re nata, ſi non ſolut. eſt alv. cras mane capt. miſt. purg. autea præſcripta.

24th. Somewhat freer from pain this morning; has had ſeveral ſtools from the bolus; went into the warm bath, and was much better after it; has taken the tinct. theb. three times.

25th. Has brought up a conſiderable quantity of matter ſince laſt night; much relieved from pain, but no relaxation of jaw. Solut. g. arab. c. ſine nitro pro potu. capt. tinct. theb. gt. xxx. pro re nata et decoct. aperient. cras mane ſi conſt. alv.

29th. Feels more pain at her ſtomach, as if there were pins and needles; ſickneſs and vomiting; jaw looſer.—Cont. pot. purg. et tinct. theb. gt. xxv. aggrediente ſpaſm.

June 2d. Yeſterday brought up a pin; ſtill feels pain and pricking at her ſtomach; coſtive.—Capt. miſt. purg. ſtatim. Cont. med.

3d. Brought up four pins together, and one ſingly before theſe.—Cap. miſt. purg. ſtatim.—Drink warm gum water.

6th. Feels no pain but ſoreneſs.—Cont. med.

9th. Complains of pain in left breaſt; apply linen dipped in Goulard's water, and ſprinkled with laudanum.

14th. Fotus of no ſervice; opening mixture does not agree. ℞. Aloe ſocotrin. rhei, ſaponis aa ʒj. ol. junip. q. s. ft. pil. xxxvi. capt. iij. conſtipat. alv.

Stomach much ſwelled and hard. ℞. Ungt. merc. fort. ℥j.

opii opii camphoræ a. ℥j. M. fricand. omni nocte et mane scrob. cordis.

17th. Much pain in her stomach; two pins or needles to be felt on each side of her stomach. Capt. opii gr. j. aggrediente dolore et rep. pro re nata. Cont. pil. aper. pro re nata. Omitt. ung. One needle taken out this day.

29th. Dismissed, relieved.

Aug. 11th. Re-admitted. On Friday last threw up a pin from her stomach; since easier there, but had pain in right breast. One needle taken out from the surface, but has continued pain from one deep seated in the same breast, with spasms, and jaw stiff. Rept. pil. purg. ut Jun. 14, præscript constipat. alv. sumend. Capt. tinct. theb. gt. xxx. hor. somni et rep. omni 3tia. hora perstante dolore et spasmo. Warm bath this evening.

16th. Spasms have been severe, and has taken the laudanum to the amount of 500 drops; took yesterday, opii gr. ij. omni 2da. hora. No pain except in breast. Cont. tinct. theb. ut antea, et pil. purg. pro re nata. Warm bath whenever the spasm or pain increases. Tinct. theb. gt. xxx. omni hora valde urgente dolore.

20th. Great pain in breast, and in jaw, which was locked; general convulsions and violent. ℞. Calomel ℈ss. opii gr. ij. muc. g. arab. q. s. ft. bol. stat. sumend. et 4ta. quaque hora repetend perstante spasmate. Warm bath ut antea.

22d. Took a bolus on Friday, went into the warm bath, and was much relieved; repeated bolus twice on Saturday, and one last night, using the warm bath. A splinter came away from the inner angle of the lower jaw on the right side; body open and jaw quite relaxed; no spasm, but violent pain in her breast. Rep. bol. et baln. calid. pro re nata.

26th. For several days has complained of great pain in her breast; and describes it to be as if several pins were lodged in the mamma and pectoral muscle, and lying between the two ribs.

Aug. 30th. The right mamma was extirpated this day, in the middle of which a needle was found closely impacted; an hæmorrhage taking place in the evening, the dressings were removed, and a small artery was taken up; a pin was found in the dressings.

Sept. 4th. Complained of pain; the dressings were partially removed, and another pin was sticking to the dressings; four other pins were also discovered in the wound, which were removed without difficulty. One of the pins having lost the head, her perception was so accurate as to distinguish it before removing the dressings.

6th.

6th. On removing the dreſſings two pins were found adhering to them. She reſted well, and has loſt every ſymptom of ſpaſm or pain, except what is in conſequence of the operation.

7th. Two more pins were found lodging on the dreſſings this day, together with a plumb ſtone which ſhe ſwallowed two days ago. No fever, but her ſtomach rejects whatever medicine is offered. Bibat aq. mephit. ſimp. pro potu.

8th. The food taken came away from the wound in the form of poultice.

9th. Complains of ſlight degree of pain in different parts of the breaſt. The wound in a healing ſtate.

10th. No material alteration.

11th. The food ſwallowed ſtill continues to work out at the wound, iſſuing from a ſinus very ſmall, and covered with granulations acting as a valve to it. Ordered to eat no animal food, or dry bread, but live only on milk diet in different forms, with ſago and rice. Port wine ℔ſs. per diem.

12th. Complains of much pain, which ſhe thinks is owing to a piece of bone working its way along the ſinus to make its exit at the wound. The piece of bone ſhe imagines is about half an inch long, and rather thick.

13th. This morning came away exactly as ſhe had deſcribed it, and appears to be part of one of the ribs; ſhe ſtill complains of much pain, and thinks there is another piece of bone making its way to come out. This evening the other piece of bone came away, covered as ſhe had deſcribed it; and appears to be the end of one of the ribs, covered with a vaſt number of inſects of the grub kind.

14th. A great number of grubs came away with the dreſsings this day

15th. A ſmall piece of bone came away with the dreſſings; complains of much pain, as if there were ſeveral more pieces to come away.

16th. This morning another piece of bone came away, rather larger than that of yeſterday.

17th. Complains of much pain and prickings in the part; food paſſes through the ſinus.

18th. Two pieces of bone came away covered with a cartilaginous ſubſtance; from the kind of pain ſhe feels, thinks there is another *large* piece of bone that will ſoon come away.

19th. This morning the large piece of bone came away as ſhe had deſcribed it; is much eaſier; almoſt every thing ſhe eats or drinks ſtill eſcapes through the ſinus.

20th. The wound looks well; no food has paſſed the ſinus to-day.

21ſt. This morning four ſmall pieces of bone came away, and and there was alſo a conſiderable quantity of food on the dreſſings; not ſo faint and low.

22d. Five ſmall pieces of bone and a quantity of food came away with the dreſſings.

23d. She is much eaſier to-day, and very little food has paſſed through the ſinus.

24th. Paſſed no food through the ſinus ſince yeſterday, and takes it freely; thinks the rib, from which the exfoliation has taken place, is now detached from the back bone.

27th. Does not take food ſo eaſily; it is heavy at her ſtomach, and occaſions nauſea, but does not paſs through the ſinus; the rib feels to her as though it were broken into ſeveral pieces. One piece of carious bone came away this day.

30th. One piece of bone came away on the 28th; could take no food; was ordered nutritive clyſters of milk and broth. Yeſterday two bones came away, portions of rib; feels as if more were lodged at the œſophagus; feels as if a gathering was coming on lower on the ribs; chillineſs and ſhivering at times; clyſter came up in part at her mouth, and gave her pain; leſſen the quantity, and repeat them. Took gt. xxx tinct. theb. laſt night, but no reſt. Rept. tinct. gt. xxx hor. 8va. et gt. xv hor. 11ma. perſtante dolore. Three pieces of carious rib came away this morning.

Oct. 1ſt. This morning *thirty-four* pieces of bone were working their way through the ſinus. Took a ſmall quantity of bread and wine laſt night, which greatly refreſhed her; eat ſome bread and milk for ſupper with ſome degree of appetite.

2d. This morning was conſiderably better; was got up, and walking about the ward in good ſpirits. After the wound was dreſſed was ſeized with ſpaſmodic affection of the lungs, almoſt producing ſuffocation, which was ſucceeded by rigor and fainting. No bone or aliment came away with the dreſſings. In the afternoon and evening was much better.

3d. Continues nearly the ſame as yeſterday.

4th. The food ſhe takes paſſes off by ſtool ſoon after ſhe has taken it; ordered to eat rice gruel as her principal diet; take from 20 to 80 drops of laudanum half an hour before ſhe eats any food.

5th. Food continues to paſs undigeſted, almoſt as ſoon as taken. The laudanum has not had any ſenſible effect.

6th. This morning a portion of bone, about three quarters of an inch in length, of a curved form with points, was diſcharged by ſtool, ſince which ſhe has been free from pain, and breathing eaſier.

7th. Took 80 drops of tinct. theb. without the leaſt effect; complains complains of frequent rigors, ſucceeded by heat, ſeveral times a day.

8th. Laſt night and this morning has taken 90 and 100 drops of tinct. theb. without any effect whatever. Can get but little reſt, from a univerſal ſoreneſs of the right ſide, which ſhe deſcribes as if her ribs were falling out of their ſockets. Omit. tinct. theb.

12th. Had nothing come away from ſore till to-day a few pieces of bone, (no *number* ſpecified in my minutes); has had cough, and expectorated dark fœtid matter, but not to-day; complains of great pain in the ſtomach, as if a large piece of bone was there; food paſſes off quick as before.

14th. This morning brought up a large piece of bone; complains of great ſoreneſs of the œſophagus; food continues to paſs unaltered, and almoſt immediately after being ſwallowed.

15th. Food ſtays longer. Cont. ſolut. gummi pro potu commune.

19th. Feels now as if pins, or a piece of bone, were penetrating the bladder, or the right ſide near the neck of the bladder.

21ſt. This day paſſed a piece of bone by urine.

25th. On Saturday and yeſterday ſhe threw up blood, liquid, not congealed, ſhe thinks about a tea-cup full. Complains of pain in her ſtomach, but not as if any extraneous ſubſtance was lodged there.

28th. Conſtant nauſea and vomiting on taking food; feels ſomething thick and long, which ſeems to come to her throat, afterwards returns to the ſtomach, and lies heavy on the left ſide of her ſtomach.

Nov. 1ſt. Symptoms as before, only yeſterday morning a red ſpot or two on the right breaſt, very ſore and inflamed; has now the appearance of an eſchar, and covers the upper part of the breaſt; no ſenſation as of pins or needles, or any irritating ſubſtance in the breaſt; frequent chillineſs and heat. Capt. julep. ſalin. ℥j. 4ta. quaque hora.

4th. Throws up food and medicines; eſchar ſloughed off; otherwiſe as before.

9th. Breaſt healing on the outſide, but yet feels pain internally, as though there were bones in it; takes food better, and it ſtays with her.

11th. Complains greatly of the heart-burn; a thin diſcoloured matter diſcharges from an ulcer in the breaſt. ℞. Miſt. cretac ℥vj. magn. alb. ʒj. M. capt. coch. j. urgente cardialgia.

15th. Cardialgia ſtill continues. ℞. Sal. ſodæ ʒij. aq. menth. pip. ℥xij. M. capt. ℥jſs. ter die. Felt as if a bone roſe

 from from her ftomach; fhe thinks the bones came from the left breaft, which is now healed excepting a fmall ulcer.

18th. Still complains of heart-burn. Rept. Mift. modo adde fal. fod. ʒj. Breaft almoft healed. Brought up two fmall bones yefterday.

20th. Complains of rib on the left fide, under the breaft, feeling as if it was fplintered; heart-burn continues. Capt. magn. alb. calcin. ℈j. cum. fing. dos. mift. falin.

23d. Had pain in her jaw, and ftiffnefs; went into the warm bath, which relieved her. Rept. baln. pro re nata. Cont. mift.

27th. Complains as before, only more pain in breaft, and vomiting; lefs cardialgia.

Dec. 1. Breaft very painful; no cardialgia; ulcer on the breaft; food ftays; body open.

8th. For the laft feven days has had almoft conftant ichorous difcharge from the breaft, with the ufual eryfipelatous appearance, attended fometimes with great ficknefs.

9th. Yefterday diarrhœa came on, with difcharge of matter. ℞. Julep. c. creta. ℔fs. pulv. c. creta. c. aa. opii ʒij. M. capt. j. poft fingulas fedes liquidas.

17th. Purging has ceafed; ftill complains of much pain in the breaft, and acid on the ftomach. Rept. mift. falin. ut antea.

23. Complains of much pain in her arm and fhoulder. App. Emp. epift. part.

26th. Complains of pain in her breaft, but much eafier fince the application of the blifter. Difmiffed, cured.

March 8, 1785. On re-admiffion fhe complains of great pain in the left eye, that fhe defcribes as proceeding from her breaft on that fide. The eye-lid much fwollen and inflamed, and one part of it has put on the appearance of efchar, that has been obferved in other parts of the body to terminate in excoriation.

11th. The right eye is now in the fame ftate as the other, and equally painful. Appl. hirud. iij. ad tempor.

14th. A confiderable quantity of blood was taken by the leeches. The fwelling and inflammation is nearly gone.

21ft. She has complained of pains in the right fide for feveral days, extending along the courfe of the right ureter, and this morning fays it has ftopped the difcharge of urine. On examination, a piece of bone was found lodged in the upper part of the vagina, on the right fide of the os uteri, and was extracted.

30th. To this time the fymptoms have remained much the fame, and five pieces of bone have been extracted at different times, times, ſince the 21ſt inſtant. N. B. One of the pieces was found making its paſſage into the vagina, at the part above mentioned, and after extraction the aperture was large enough to admit the point of a finger.

(From this report I do not find any minute until)

Oct. 18, Diſmiſſed, cured.

Jan. 3. Re-admitted; fell down ſtairs; head and ſide much bruiſed; ordered to have the head ſhaved about the part affected, and covered with empl. neutrale. ℞. Infus. ſen. ℥iij. tinct. ſennæ ℥j. ſal. cath. amar. Mannæ aa. ℥j. tinct. jalap ʒſs. M. capt. ℥iſs. 4ta. quaque hora donec alvus ter depon.

℞. Vin. antimonial, tinct. theb. aa. ʒij. M. capt. coch. j. parv. hora ſomni exhauſtu commun.

Feb. 3. Extracted four bones from the vagina, the whole about an inch long, to appearance exfoliations of the ribs. Complains of great pains in the ſide, as if more bones were making their way downwards. Cutaneous ulcerations of the legs.

℞. Julep. card. ℔ſs. capt. ℥j. 3tia. vel 4ta. quaque hora languoribus. Capt. tinct. theb. gt. xxx. h. ſ. ex julep. card. ʒjſs. et repet. gt. xx. poſt horas 4tas dolor urgente. Bowels open; makes water.

4th. Extracted three bones laying acroſs, and conſiderably farther within the vagina. The laſt bone ſeemed to be retained about half its length within the ſinus, from which they are ſuppoſed to make their exit at the lower part between the os uteri and rectum. Great pains of ſide and ſtomach; alternate chills and fluſhing; tongue white and furred; no appetite. Capt. tinct. theb. gt. xxx. et vin. antim. gt. xx. ex ſingulis doſibus, repet. julep. card. pro re nata ut antea.

17th. After making water complains of lacerating pain of the right ſide, as if pieces of bone were ſtill moving downwards to the vagina, and having alternate ſymptoms of pyrexia, ſickneſs, pain at the ſtomach, &c. with hardneſs and ſwelling externally, where ſhe ſuppoſes another needle to be deep ſeated; ſhe thinks ſhe can fully diſtinguiſh its form and points. Several pieces of bone of unequal ſizes, ſome one inch in length, and half an inch in breadth, (about twelve in number) in their appearance like a divided portion of rib, putrid, blackiſh, and covered with offenſive matter, ſome of them partially remaining within the ſinus, betwixt the rectum and uterus, others looſe within the vagina, have been extracted at different times ſince the 4th.

Upon examining the matter brought up by vomiting, ſome ſmall inſects were obſerved in the maſs, which was of a darkiſh colour, very low and weak.

 18th.

18th. Still complains of violent pain, as if more bones were paſſing. Extracted three pieces; within the middle of one portion (to appearance rib) of about ¼ of an inch in length and ½ an inch in breadth, a large pin was found running longitudinally through part of its ſubſtance, and firmly impacted. She thinks more bones are working downwards the ſame way.

19th. Extracted a very ſmall needle from the fore part of the leg, near the outer ancle; and from the deep-ſeated pain near the ſame part, ſhe fancies a pin is lodged. The eryſipelatous ulceration, which affected the greateſt part of the leg, has entirely gone off.

20th. Have extracted another portion of bone, nearly one inch in length and ½ an inch broad, from out of the vagina, having two pins running parallel to each other, in the ſame manner as in that of the report of the 18th. Feels more bone. The former ſymptoms ſtill continue; ancles œdematous at night; cannot ſleep for pain, or retain any ſolid food whatever. Cont. julep. card. et tinct. thebaic.

25th. Complains of very lacerating, pricking pain all laſt night. Have removed a portion of bone, having a pin running through its ſubſtance longitudinally; and another portion, ¼ of an inch in length and ½ an inch in breadth, with a pin running tranſverſely through it, forming a right angle. Had a ſtool, which was ſtreaked with blood; a continued diſcharge of pus, black and fœtid, from the vagina; ſtill complains of pain in the ſide, ſtomach, &c. with ſickneſs as before.

March 3d. Removed two narrow portions of bone, about 1 inch in length and ¼ inch in breadth; one portion having two pins impacted longitudinally, the other only one. Thinks the whole of the looſened bones from the ribs are diſcharged, as ſhe is free from lacerating pains; other ſymptoms continue; gets little or no ſleep.

5th. Complains of pain in the bowels, as if a bone was paſſing through them; ſtools very black; ſeveral of them in a day, with grubs, the ſame as in the matter vomited before. The ſtools ordered to be ſaved for examination.

8th. Upon examination the ſtools were found very black, and a pin was diſcovered at the bottom of the veſſel, diſcoloured, and ſeemingly in part diſſolved by a menſtruum.

10th. Shortneſs of breath when lying; the legs, thighs, and ſkin of the breaſt œdematous; great diſtention and hardneſs of the ſtomach and abdomen; has paſſed no urine theſe three days. Capt. ſpt. nitri dulcis cochl. min. j. 3tia. vel 4ta. quaque hora. The nitrous ſpirit cauſed vomiting, by which a quantity of ſaline taſted water was ejected from the ſtomach. Now more eaſy.

11th,

11th. One quart of water was drawn off by the catheter.

14th. Feels very great fulnefs with load at ftomach; thinks no urine is fecreted into the bladder; has not made water thefe three days, nor has any inclination; has chilly fits fucceeded by heat two or three times a day; has ftools daily, of a dark colour.

18th. Œdema, and tightnefs about præcordia not fo confiderable; complains of great pain in the bladder, efpecially during the excretion of urine, which is now more frequent; pain of the right leg from a pin.

22d. Has complained for three days paft of pain in her bowels, with purging to the extent of nine, ten, or eleven ftools a day, with great pain in making water. The urine depofits a copious earthy fediment, mixed with mucus of a greyifh caft.

℞. Aq. cinnam. ij. puræ ℥ivfs. tinct. kino ʒj. theb. ʒfs. tinct. cardamom. ʒfs. M. capt. ʒfs. poft fingulas fedes liquidas, vel pro re nata dolor inteftinorum.

24th. Purging continues; makes very little water, and then with great pain; in the night of the 22d had a very ftrong convulfion fit, which threw her out of bed, and bruifed her very much; has had frequent vomitings, as before; complains of great pains in the bones of the facrum and loins; the ftomach not fo much fwelled or hard.

℞. Sal. polychreft pulv. rhei aa. gr. v. M. capiat nocte maneque. Solution of gum Arabic for common drink, with red wine.

℞. Ol. camphor. ℥ij. tinct. theb. ʒfs. ft. lin. ter in die ad part dolor. affect.

April 20th. Violent pain in her bowels with continual purging; has difcharged two pins with fome fragments of bone by ftool; pain as ufual in her right fide.

21ft. Extracted fome portions of bone (no number mentioned) from the finus in the vagina, through one of which a pin was driven.

30th. Since the 21ft, eafier in her fide, bowels, and back.

May 1ft. Great pain in her right breaft, pricking as if feveral pins were buried deep; the glandular parts enlarged and hard; can feel two or three feemingly buried in the middle of thefe indurated glands; takes fix or feven tea fpoons full of tinct. theb. two or three times a day without any effect either in producing fleep or mitigating pain; had feveral convulfion fits this night, which were only relieved by large dofes of opium. (N. B. Each tea fpoonful held 100 drops.)

4th. Pain this evening intolerable in her breaft; took at eleven in the morning, *eleven tea spoons full* of tinct. thebaica, and and this morning *twelve*, which have not yet (eight o'clock) either alleviated the pain or produced any effect, except exciting nausea. ℞. Opii, Castor, Ruff. aa. ℈j. M. et divide in pil. xxiv. capt. ij. 3tia. quaque hora dolore perstante.

(From this day we find no report until)

June 12th. Dismissed, cured.

July 26, 1792. I have this day been credibly informed by a neighbour and relation of Kate Hudson, that she is married, has two fine children, and enjoys better health than for several years past.

At present, I shall make no Comment on the Case; I feel it, however, a duty I owe to myself, (and to anticipate the attacks of *puny* Criticism) that I should here observe, that the language of the Case throughout, is strictly that of the minutes preserved in the Case Books of the Hospital, as taken thence by myself upwards of ten years ago.

HUGH MOISES, M. D.

